# JIB-04, a histone demethylase Jumonji C domain inhibitor, regulates phenotypic switching of vascular smooth muscle cells

**DOI:** 10.1186/s13148-022-01321-8

**Published:** 2022-08-13

**Authors:** Yi He, Xin Yi, Zihao Zhang, Hanshen Luo, Rui Li, Xin Feng, Ze-Min Fang, Xue-Hai Zhu, Wenlin Cheng, Ding-Sheng Jiang, Fang Zhao, Xiang Wei

**Affiliations:** 1 Division of Cardiothoracic and Vascular Surgery, Sino-Swiss Heart-Lung Transplantation Institute, Tongji Hospital, Tongji Medical College, Huazhong University of Science and Technology, 1095 Jiefang Ave, Wuhan, 430030 Hubei China; 2 Department of Cardiology, Renmin Hospital of Wuhan University, Wuhan, China; 3 Key Laboratory of Organ Transplantation, Ministry of Education, NHC Key Laboratory of Organ Transplantation; Key Laboratory of Organ Transplantation, Chinese Academy of Medical Sciences, Wuhan, Hubei China; 4 Department of Cardiology, Zhongnan Hospital of Wuhan University, East Lake Road 169, Wuhan, Hubei China; 5 Institute of Myocardial Injury and Repair, Wuhan University, Wuhan, Hubei China

**Keywords:** Neointima formation, Histone demethylase, JMJDs, Vascular smooth muscle cell, Phenotypic switching, Autophagy, JIB-04

## Abstract

**Background:**

Vascular smooth muscle cell (VSMC) phenotype switching is critical for neointima formation, which is the major cause of restenosis after stenting or coronary artery bypass grafting. However, the epigenetic mechanisms regulating phenotype switching of VSMCs, especially histone methylation, are not well understood. As a main component of histone lysine demethylases, Jumonji demethylases might be involved in VSMC phenotype switching and neointima formation.

**Methods and results:**

A mouse carotid injury model and VSMC proliferation model were constructed to investigate the relationship between histone methylation of H3K36 (downstream target molecule of Jumonji demethylase) and neointima formation. We found that the methylation levels of H3K36 negatively correlated with VSMC proliferation and neointima formation. Next, we revealed that JIB-04 (a pan-inhibitor of the Jumonji demethylase superfamily) could increase the methylation levels of H3K36. Furthermore, we found that JIB-04 obviously inhibited HASMC proliferation, and a cell cycle assay showed that JIB-04 caused G2/M phase arrest in HASMCs by inhibiting the phosphorylation of RB and CDC2 and promoting the phosphorylation of CHK1. Moreover, JIB-04 inhibited the expression of MMP2 to suppress the migration of HASMCs and repressed the expression of contraction-related genes. RNA sequencing analysis showed that the biological processes associated with the cell cycle and autophagy were enriched by using Gene Ontology analysis after HASMCs were treated with JIB-04. Furthermore, we demonstrated that JIB-04 impairs autophagic flux by downregulating STX17 and RAB7 expression to inhibit the fusion of autophagosomes and lysosomes.

**Conclusion:**

JIB-04 suppresses the proliferation, migration, and contractile phenotype of HASMCs by inhibiting autophagic flux, which indicates that JIB-04 is a promising reagent for the treatment of neointima formation.

**Supplementary Information:**

The online version contains supplementary material available at 10.1186/s13148-022-01321-8.

## Introduction

Cardiovascular diseases (CVDs) are the leading cause of death worldwide, the prevalence and mortality of which were approximately 485.6 million cases and 17.8 million cases in 2017, respectively [1]. Coronary atherosclerotic heart disease (CHD) is the dominant cause of death and accounts for 42.6% of all cases. Percutaneous coronary interventions (PCIs) and coronary artery bypass grafting (CABG) are well-established therapeutic options for revascularization of CHD [2, 3]. Although PCI is safe and shows good short-term efficacy, stent restenosis, a primary complication with an occurrence ranging from 20 to 50%, restricts the long-term efficacy of PCI. In-stent restenosis is mainly caused by neointima formation, which is induced by several vascular injury procedures [4]. Similarly, obstruction of vein graft after surgery also limits the efficacy of CABG. Early obstruction of vein grafts in the months after surgery is relevant to endothelial damage and consequent thrombosis, whereas late obstruction of vein grafts in the years after surgery is connected with atherosclerosis caused by neointima formation [4]. Hence, it is vital to inhibit neointima formation to attenuate restenosis after PCI and graft obstruction after CABG to improve the outcome of CHD patients.

The proliferation, migration, and phenotypic switching from contractile to synthetic phenotypes of vascular smooth muscle cells (VSMCs) are critical for neointima formation and vascular restenosis [5]. Recently, histone methylation was reported to participate in vascular biology, including neointima formation [6]. Our previous studies have shown that the histone methyltransferases EZH2 and EHMT2 affect VSMC survival by regulating autophagy [7, 8]. Given that histone methylation is reversibly modified by histone demethylases and methylation modifications at different sites of histones have different functions [9, 10], it is critical to investigate the roles of histone demethylases in neointima formation.

The Jumonji C domain-containing protein (JMJD) family, a main component of histone lysine demethylases (KDMs), are oxygenases that achieve demethylation through hydroxylation reactions. JMJD demethylases catalyze α-ketoglutarate (αKG), oxygen, and Fe(II) to produce succinate and CO_2_ in the hydroxylation reaction, in which unstable hemiaminal products are produced and broken into formaldehyde and a demethylated histone product [11, 12]. Recently, demethylase KDM3a was reported to be upregulated during neointimal hyperplasia after vascular injury in diabetic rats [13], and demethylase JMJD3 (KDM6B) and KDM1A were found to be highly expressed in a rat carotid artery balloon injury model [5, 13]. These results indicated that KDMs might be involved in neointima formation.

In the present study, to explore the roles and mechanisms of KDMs in neointima formation, we used JIB-04, a pan-inhibitor of JMJDs, to treat human aortic smooth muscle cells (HASMCs). We found that JIB-04 inhibited the proliferation, migration, and contractile phenotype of HASMCs by regulating H3K36 methylation. More importantly, we revealed that JIB-04 impaired the fusion of autophagosomes and lysosomes by downregulating STX17 and RAB7 expression to affect the phenotypic switching of HASMCs. Thus, our results indicated that JIB-04 might be a promising drug for inhibiting neointima formation and improving restenosis after CABG or stenting.

## Results

### The target histone methylation levels of JMJDs were associated with VSMC proliferation and neointima formation

**Fig. 1:**
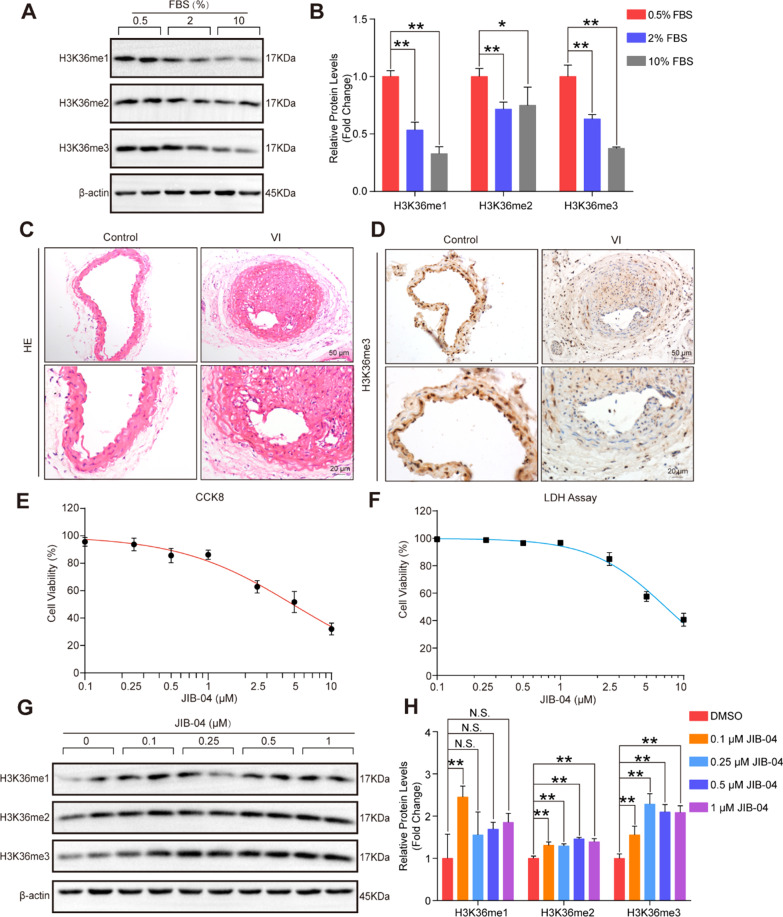
H3K36me3 was negatively correlated with HASMC proliferation and neointima formation. **A-B**. Western blot analysis showed the protein expression of H3K36me1, H3K36me2, and H3K36me3 in HASMCs cultivated with 0.5%, 2%, and 10% fetal bovine serum (FBS) (*n* = 4 per group). β-Actin served as a loading control. *P < 0.05, **P < 0.01. **C.** Representative images of H&E staining of carotid arteries in the control group and vascular injury (VI) group. Scale bar: 20 and 50 μm. **D.** Representative images of immunohistochemical staining of H3K36me3 in the carotid arteries. H3K36me3-labeled nuclei are shown in brown. Scale bar: 20 and 50 μm. **E.** Cell viability of HASMCs after treatment with different concentrations of JIB-04 (0, 0.1, 0.25, 0.5, 1, 2.5, 5, and 10 μM) (*n* = 5 per group). **F.** The toxicity of different concentrations of JIB-04 (0, 0.1, 0.25, 0.5, 1, 2.5, 5, and 10 μM) on HASMCs was evaluated by LDH release levels (*n* = 5 per group). **G-H**. H3K36me1, H3K36me2, and H3K36me3 protein levels in HAMSCs treated with different concentrations of JIB-04 (0, 0.1, 0.25, 0.5, and 1 μM) were measured by western blotting (*n* = 4 per group). β-Actin served as a loading control. *P < 0.05, **P < 0.01, N.S. no significant

We first established a HASMC proliferation model by stimulating cells with different concentrations of fetal bovine serum (FBS). We further detected the methylation levels of H3K4, H3K9, and H3K36, which were potential targets of JMJDs, in the HASMC proliferation model. The results showed that compared with that of the 0.5% FBS group, the mono-, di-, and trimethylation of H3K36 was decreased in the HASMCs treated with 2% or 10% FBS (Fig. 1A and B). Similarly, the mono-, di- and trimethylation of H3K9 were decreased in the HASMCs treated with 10% FBS (Additional file 1: Figure S1A and B). Furthermore, the di- and trimethylation, rather than monomethylation of H3K4 were decreased in the HASMCs treated with 2% or 10% FBS (Additional file 1: Figure S1C and D). To further confirm whether the methylation of H3K4, H3K9, and H3K36 was associated with neointima formation, we established a mouse model of neointima formation through carotid artery injury for 28 days. The results of H&E staining demonstrated that compared with the controls, the injured carotid showed an obviously narrowed vascular cavity and thickened intima (Fig. 1C), suggesting that the mouse model was successfully constructed. We further detected the expression of H3K36me3 in injured arteries and control arteries by immunohistochemistry, and the results showed that H3K36me3 was significantly decreased in the injured carotid arteries compared to the control arteries (Fig. 1D). In addition, H3K4me3 was significantly decreased, while H3K9me3 was markedly increased in the injured carotid arteries compared to the control arteries (Additional file 1: Figure S1E and F). Thus, these results indicated that the methylation of H3K4, H3K9, and H3K36 may be involved in HASMC growth and neointima formation.

### JIB-04 increased the methylation levels of H3K36 in HASMCs

Since the methylation of H3K4, H3K9, and H3K36 is correlated with VSMC proliferation and neointima formation, regulating the methylation levels of H3K4, H3K9, and H3K36 can be expected to inhibit VSMC proliferation and neointima formation. Given that H3K4, H3K9, and H3K36 are demethylated by JMJDs, specific inhibition of JMJDs can enhance H3K4, H3K9, and H3K36 methylation [14]. JIB-04 is a specific inhibitor of JMJDs [15], and its role in VSMC proliferation and neointima formation remains unknown.

To explore the effect of JIB-04 on HASMCs and determine the optimal drug concentration, we first treated HASMCs with different concentrations of JIB-04 (0, 0.1, 0.25, 0.5, 1, 2.5, 5, 10 μmol/L) and evaluated cell viability by using CCK-8 and LDH assays. The results showed that JIB-04 had a dose-dependent effect on the viability of HASMCs and had little toxicity to HASMCs at concentrations below 1 μmol/L (Fig. 1E and 1F). Thus, we treated HASCMs with 0, 0.1, 0.25, 0.5, or 1 μmol/L JIB-04 to verify its effects on the methylation of H3K4, H3K9, and H3K36, which were reported to be demethylated by JMJDs [16]. Our results revealed that the protein levels of H3K36me2 and H3K36me3 (Fig. 1G and H) were significantly increased by JIB-04 treatment, and the effective concentration was as low as 0.1 μmol/L. In addition, H3K9me1, H3K9me2, and H3K9me3 were significantly increased (Additional file 2: Figure S2A and B), while no obvious change in the methylation of H3K4 was detected in HASMCs with the treatment of JIB-04 (Additional file 2: Figure S2C and D). Since 0.5 μmol/L and 1 μmol/L JIB-04 had comparable effects on H3K4, H3K9, and H3K36 methylation and to avoid cytotoxicity, we chose 0.5 μmol/L JIB-04 for subsequent experiments.

### JIB-04 suppressed the proliferation of HASMCs by arresting cells at the G2/M phase

**Fig. 2:**
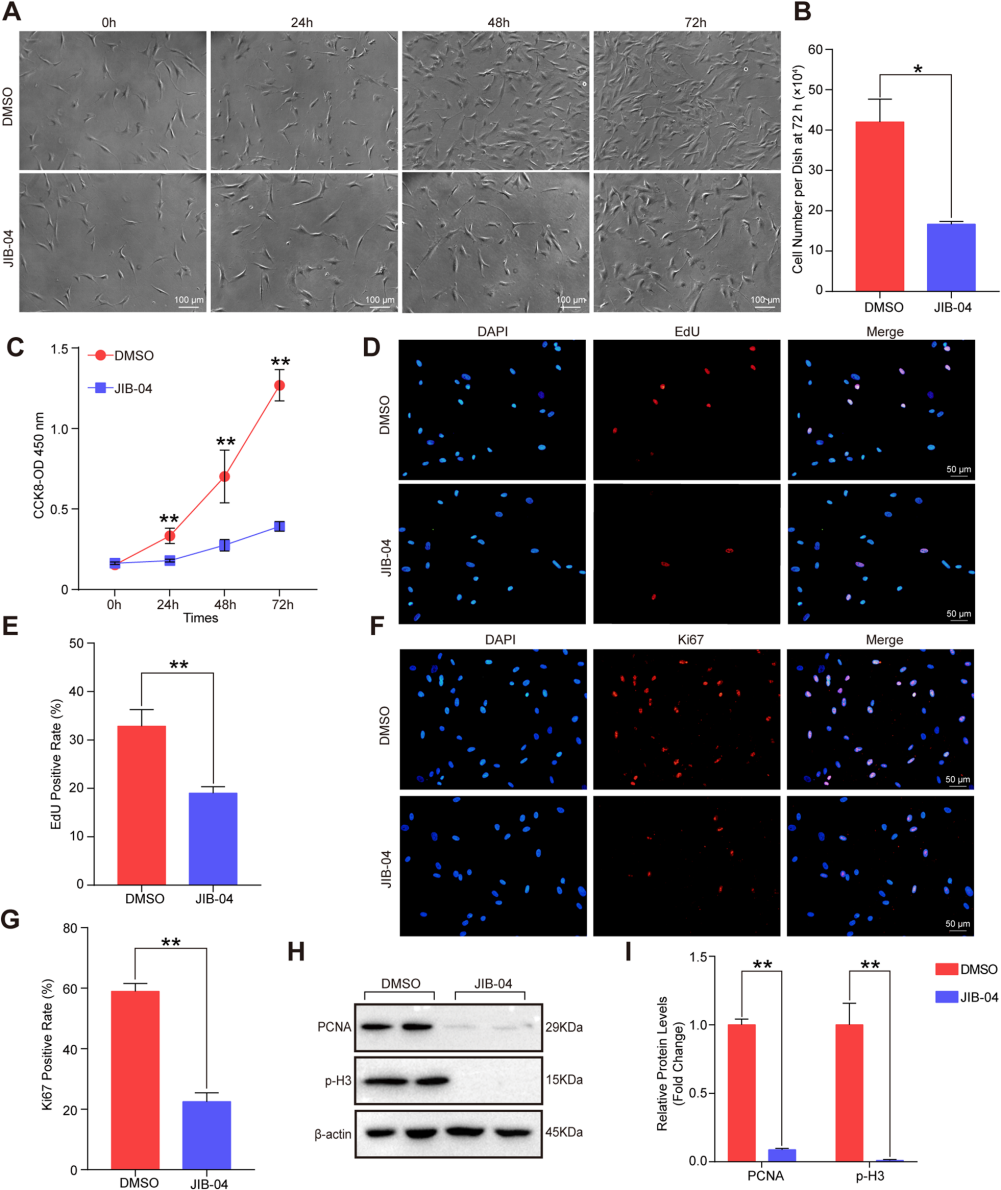
JIB-04 inhibited HASMC viability and proliferation. **A**. Microscopic observation of HASMCs in 6-cm dishes after treatment with DMSO or JIB-04 (0.5 μM) for 0, 24, 48, and 72 h. Scale bar, 100 μm. **B**. Cell counts of HASMCs in 6-cm dishes after treatment with DMSO or JIB-04 (0.5 μM) for 72 h (*n* = 3 per group). **C**. Absorbance at 450 nm of HASMCs after treatment with DMSO or JIB-04 (0.5 μM) for 0, 24, 48, and 72 h (*n* = 5 per group). **D-E**. The EdU incorporation assay showed the proliferative potential in HASMCs after treatment with DMSO or JIB-04 (0.5 μM) for 24 h, in which nuclei were stained with DAPI (blue) and EdU incorporation appeared in red. The positive EdU rate was measured (*n* = 3 per group). Scale bar, 50 μm. **F-G**. Immunofluorescence staining of proliferating nuclear antigen Ki67 revealed the proliferative capacity of HASMCs after treatment with DMSO or JIB-04 (0.5 μM) for 24 h. Nuclei were stained with DAPI (blue), and Ki67 was stained red (*n* = 3 per group). Scale bar, 50 μm. **H-I,** Western blot showed the proliferation-related protein levels of PCNA and phosphorylated H3 (p-H3) in HASMCs after treatment with DMSO or JIB-04 (0.5 μM) for 48 h (*n* = 4 per group). β-Actin served as a loading control. *P < 0.05, **P < 0.01

To investigate whether JIB-04 affects the proliferation of HASMCs, we treated HASMCs with 0.5 μmol/L JIB-04 or DMSO for 72 h. From the cell images under the light microscope in Fig. 2A, we found that the number of HASMCs was significantly reduced after JIB-04 treatment compared with DMSO treatment, and cell counts also validated this result (Fig. 2B). The CCK-8 assay further verified that JIB-04 inhibited the proliferation of HASMCs in a time-dependent manner (Fig. 2C). The EdU incorporation assay can be used to monitor DNA replication during cell division, and the results showed that JIB-04 obviously inhibited DNA replication in HASMCs (Fig. 2D and E). The positive rate of Ki67 (proliferation-related antigen) was notably reduced in the HASMCs treated with JIB-04 compared with DMSO (Fig. 2F and G). Furthermore, the markers of proliferation, PCNA, and phosphorylation of histone H3 (p-H3) were inhibited by JIB-04 (Fig. 2H and I).

**Fig. 3:**
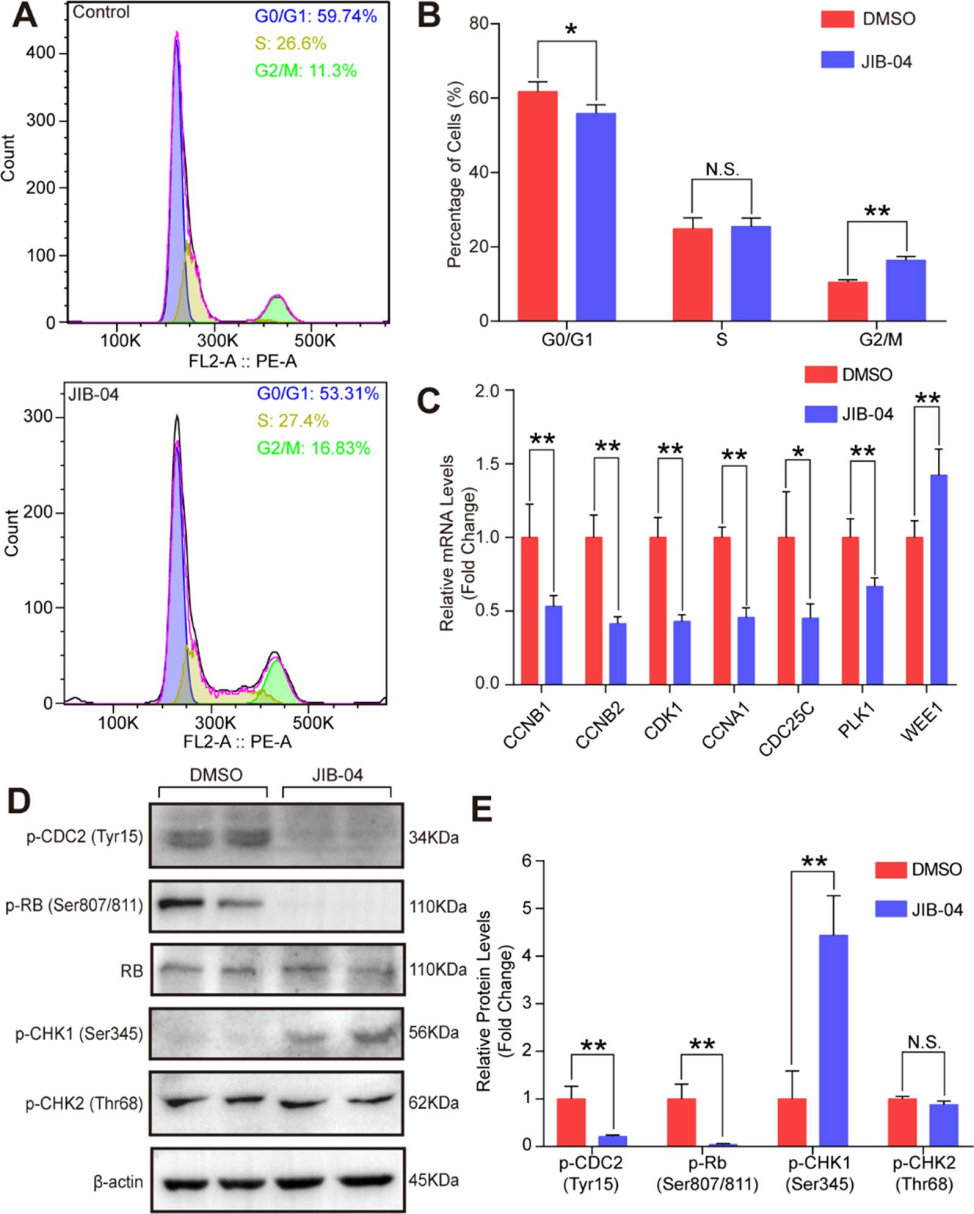
JIB-04 regulated G2/M phase gene expression and induced cell cycle arrest. **A-B**. Flow cytometry analysis showed the ratio of HASMCs in different cell cycle phases after treatment with DMSO or JIB-04 (0.5 μM) for 48 h (*n* = 3 per group). **C**. Quantification of the mRNA levels of CCNB1, CCNB2, CDK1, CCNA1, CDC25C, PLK1, and WEE1 in HASMCs after JIB-04 treatment (*n* = 4 per group). **D-E**. Protein levels of phosphorylated CDC2 (p-CDC2), phosphorylated RB (p-RB), phosphorylated CHK1 (p-CHK1), and phosphorylated CHK2 (p-CHK2) in HASMCs after JIB-04 treatment (*n* = 4 per group). β-Actin served as a loading control. *P < 0.05, **P < 0.01, N.S. no significant

Since cell division is controlled by cell cycle checkpoints, we investigated which checkpoint was inhibited by JIB-04 to affect cell proliferation. Thus, flow cytometry was performed to detect the cells in each cell phase. The results demonstrated that compared with DMSO treatment, JIB-04 treatment resulted in more HASMCs arrested in G2/M phase (Fig. 3A and B). The checkpoint of G2/M phase is regulated by many genes, and we found that JIB-04 significantly inhibited the expression of the G2/M phase-related genes CDK1, CDC25C, CCNB1, CCNB2, and PLK1 but upregulated its suppressor WEE1 (Fig. 3C). Furthermore, the phosphorylation of CDC2 and RB and dephosphorylation of CHK1 and CHK2 are critical for the G2/M checkpoint, and our results demonstrated that JIB-04 treatment obviously inhibited the phosphorylation of CDC2 and RB and facilitated CHK1 but not CHK2 phosphorylation (Fig. 3D and E). Therefore, these results indicated that JIB-04 inhibited HASMC proliferation by arresting HASMCs at the G2/M phase.

### JIB-04 inhibited HASMC migration and contractile phenotype

**Fig. 4:**
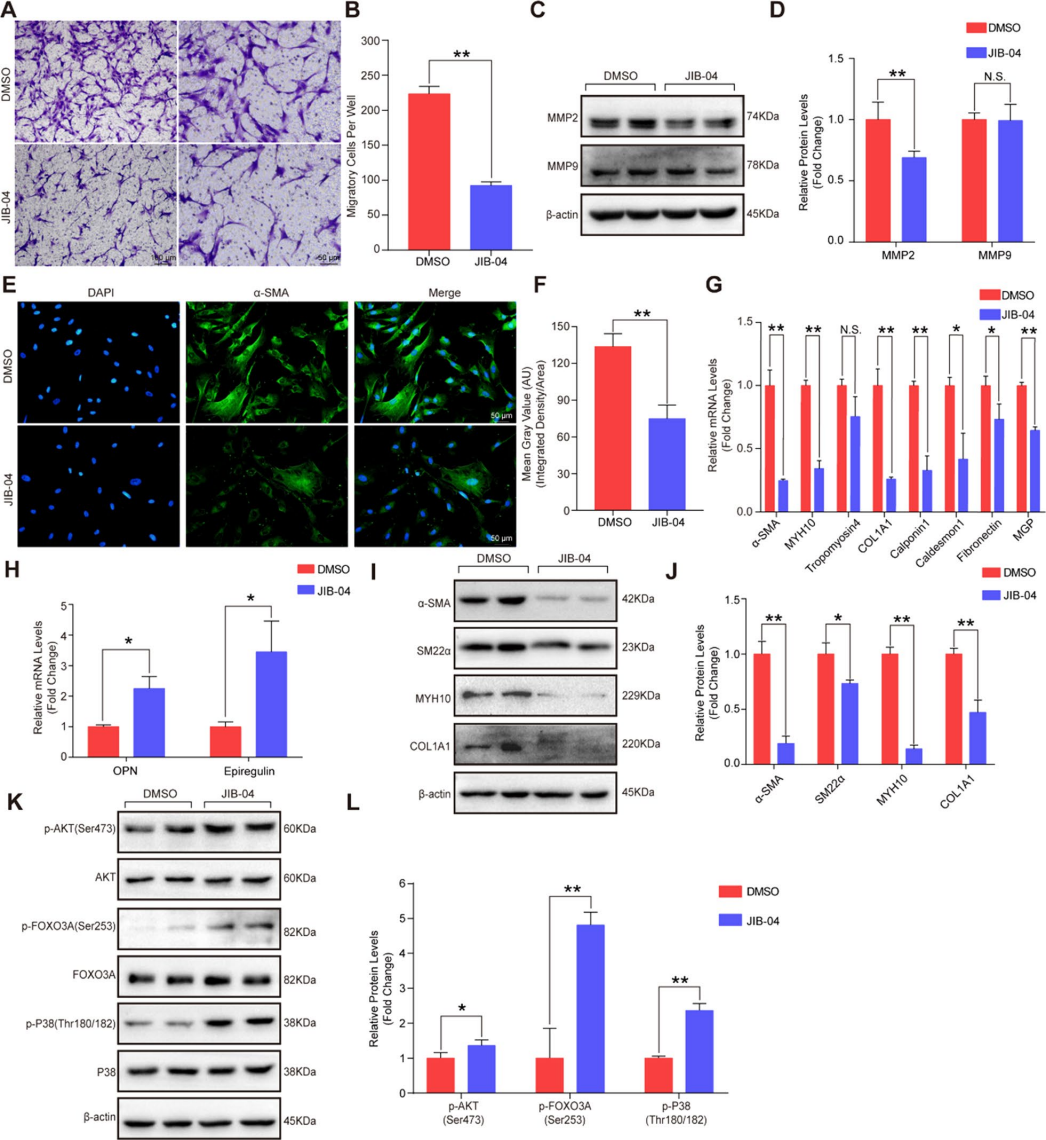
JIB-04 inhibited HASMC migration and contractile phenotypes. **A-B**. The migration of HAVSMCs was measured by transwell assays. HASMCs were treated with DMSO or JIB-04 (0.5 μM) for 24 h, and representative images of migrated cells were obtained under a microscope. Scale bar, 100 μm; Scale bar, 50 μm. Quantitative analysis of the transwell assay (*n* = 3 per group). **C-D**. The protein expression levels of MMP2 and MMP9 were detected by western blotting (*n* = 4 per group). **E–F**. The contractile phenotype was evaluated by α-SMA immunofluorescence staining, in which nuclei were stained with DAPI (blue) and α-SMA was stained in green (*n* = 3 per group). Quantitative analysis of α-SMA fluorescence intensity was performed by ImageJ (version 1.8.0). **G**. Quantification of the mRNA levels of α-SMA, MYH10, Tropomyosin 4, COL1A1, Calponin 1, Caldesmon 1, Fibronectin, and Matrix Gla protein (MGP) in HASMCs after JIB-04 treatment (*n* = 3 per group). **H**. Quantification of osteopontin (OPN) and epiregulin mRNA levels in HASMCs after JIB-04 treatment (*n* = 3 per group). **I-J**. Western blot showed the protein levels of α-SMA, SM22α, MYH10, and COL1A1 in HASMCs after treatment with DMSO or JIB-04 (0.5 μM) for 48 h (*n* = 4 per group). **K-L**. Western blot analysis showed the protein levels of phosphorylated AKT (p-AKT), phosphorylated FOXO3A (p-FOXO3A), and phosphorylated P38 (p-P38) in HASMCs after treatment with DMSO or JIB-04 (0.5 μM) for 48 h (*n* = 4 per group). β-Actin served as a loading control. *P < 0.05, **P < 0.01, N.S. no significant

Since changes in the proliferation of VSMCs are often accompanied by alterations in migration and phenotypic switching [17, 18], we further investigated the effects of JIB-04 on the migration and phenotype of HASMCs. As shown in Fig. 4A and 4B, treatment with JIB-04 for 24 h significantly decreased the migration of HASMCs. MMP2 and MMP9 have been reported to be closely associated with the migration of VSMCs [19]. We found that treatment with JIB-04 inhibited the expression of MMP2 but not MMP9 (Fig. 4C and D), suggesting that JIB-04 suppressed HASMC migration probably by downregulating the expression of MMP2.

Immunofluorescence staining showed that alpha-smooth muscle actin (α-SMA) was significantly downregulated in the HASMCs treated with JIB-04 (Fig. 4E and F). The results of real-time PCR demonstrated that contraction-related genes (α-SMA, calponin 1, caldesmon 1, MYH10, COL1A1, Tropomyosin 4, and Fibronectin) were decreased, while synthetic genes (osteopontin and epiregulin) were increased in the HASMCs treated with JIB-04 (Fig. 4G and H). In accordance with the mRNA levels, the protein levels of α-SMA, SM22α, MYH10, and COL1A1 were decreased by JIB-04 in HASMCs (Fig. 4I and J). These results suggested that JIB-04 could inhibit the migration and contractile phenotype. To further explore the potential mechanisms of JIB-04 on HASMC phenotype switching, we assessed the PI3K-AKT signaling pathway and MAPK signaling pathway, which were reported to be critical in phenotype switching of VSMCs. As shown in Fig. 4K and 4L, the protein levels of p-AKT, p-FOXO3A, and p-P38 were significantly increased in the HASMCs treated with JIB-04, suggesting that JIB-04 might promote the switch from a contractile phenotype to a synthetic phenotype by regulating the AKT-FOXO3A signaling pathway and the P38 MAPK signaling pathway.

### Transcriptome analysis indicated that JIB-04 influenced the mitotic process and induced cell cycle arrest

**Fig. 5:**
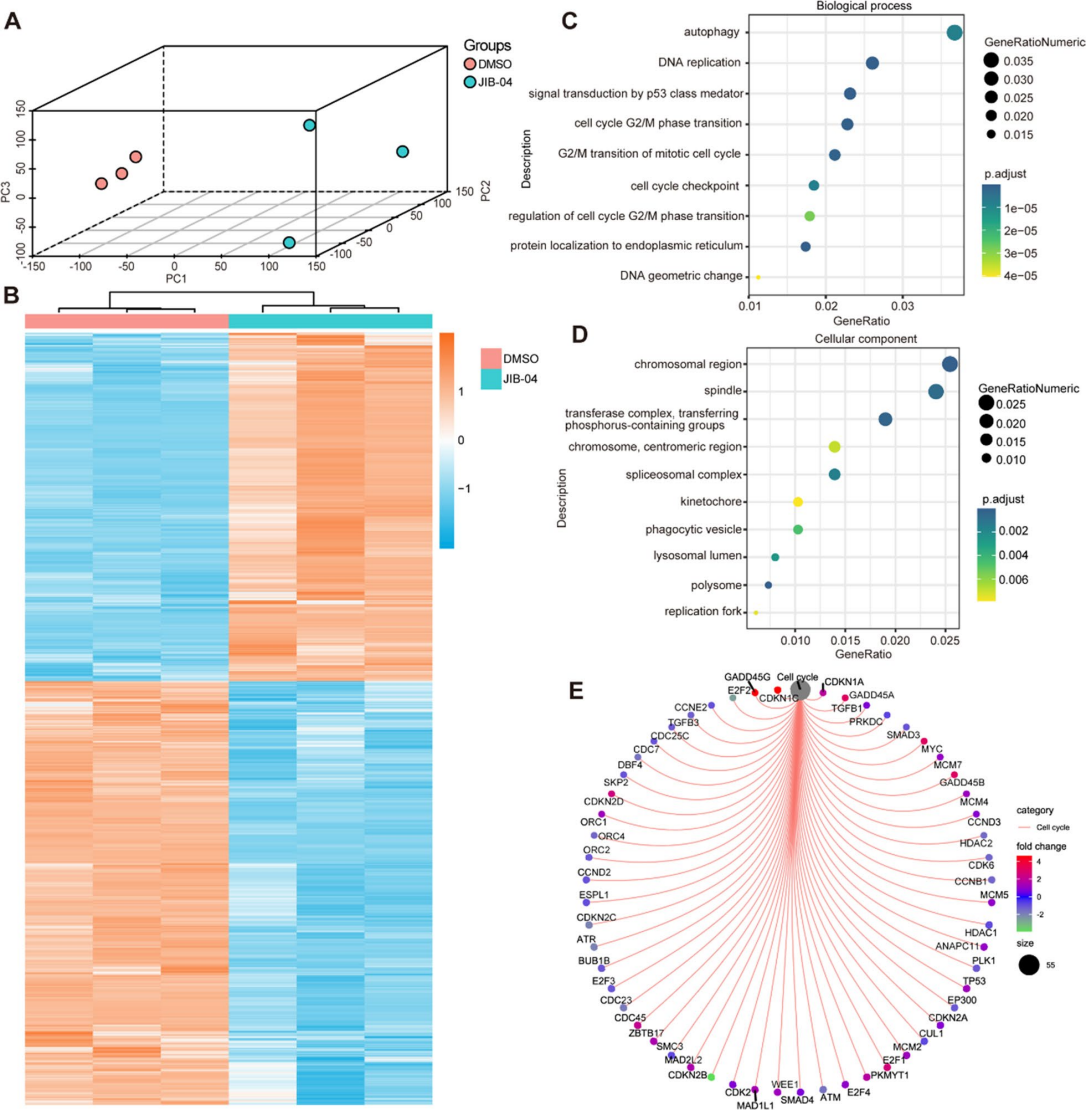
JIB-04 influenced the mitotic process and induced cell cycle arrest. **A**. Principal component analysis (PCA) to display the gene expression distribution among the DMSO and JIB-04 groups. **B**. Differentially expressed genes (DEGs) exhibited by the heatmap were markedly changed after JIB-04 treatment compared to DMSO treatment. **C-D**. Gene Ontology (GO) enrichment analysis showed that biological processes and cellular components were associated with these DEGs. **E**. Kyoto Encyclopedia of Genes and Genomes (KEGG) enrichment analysis showed different cell cycle-related gene expression patterns in the JIB-04 versus DMSO groups

To further investigate the mechanism by which JIB-04 affects HASMC phenotype switching, we performed RNA sequencing analysis of the HASMCs treated with JIB-04 or DMSO. Principal component analysis (PCA) showed clear clustering of the samples for the two groups (Fig. 5A). According to the threshold (|log2 (fold change)|≥ 0.7 and false discovery rate (FDR) < 0.05), we identified a total of 7052 genes differentially expressed between the JIB-04 and DMSO groups (Fig. 5B). Gene Ontology (GO) annotation revealed that biological processes involved in autophagy, DNA replication, and the cell cycle were highlighted (Fig. 5C), and mitosis-related cellular components (e.g., mitosis spindle, spliceosomal complex, and kinetochore) were enriched (Fig. 5D). Furthermore, the differentially expressed genes enriched in the cell cycle pathway are shown in Fig. 5E. These results indicated that the proliferative inhibition of JIB-04 might function primarily by downregulating cyclin B-CDK1 and PLK1, which disturbed the mitotic process and induced cell arrest at G2/M phase.

### JIB-04 impaired autophagic flux by inhibiting the expression of STX17 and RAB7

**Fig. 6:**
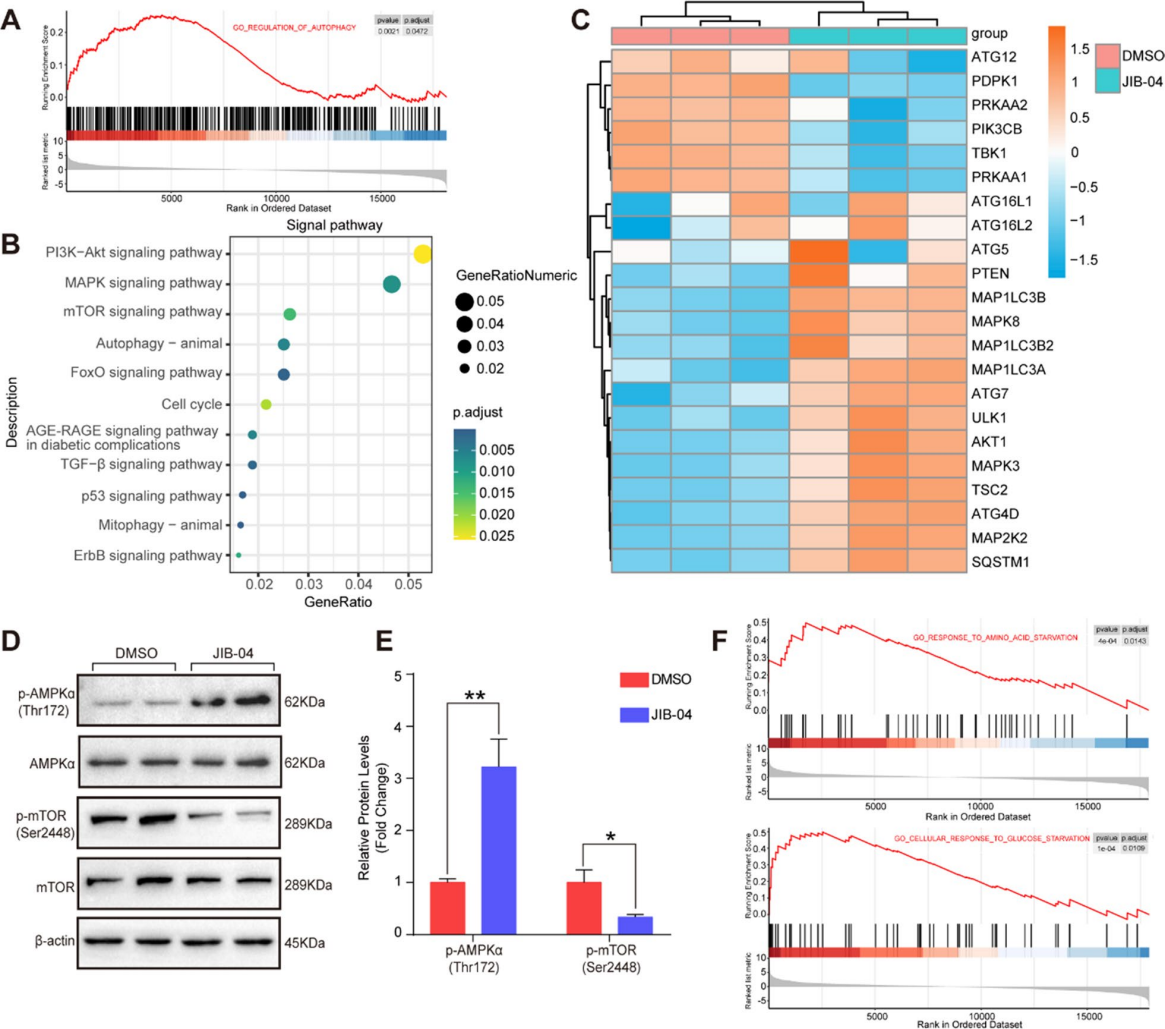
JIB-04 regulated autophagy-related SQSTM1 expression by H3K36 methylation. **A-B**. Gene Set Enrichment Analysis (GSEA) and KEGG enrichment analysis showed autophagy and autophagy-related pathway changes in the JIB-04 group compared to the DMSO group. **C**. Differentially expressed genes in the autophagy pathway are shown in the heatmap. **D-E**. Protein levels of phosphorylated AMPKα (p-AMPKα) and phosphorylated mTOR (p-mTOR) were determined by western blotting (n = 4 per group). β-Actin served as a loading control. **F**. GSEA showed the response of HASMCs to glucose and amino acid starvation in the JIB-04 vs. DMSO group. *P < 0.05, **P < 0.01

**Fig. 7:**
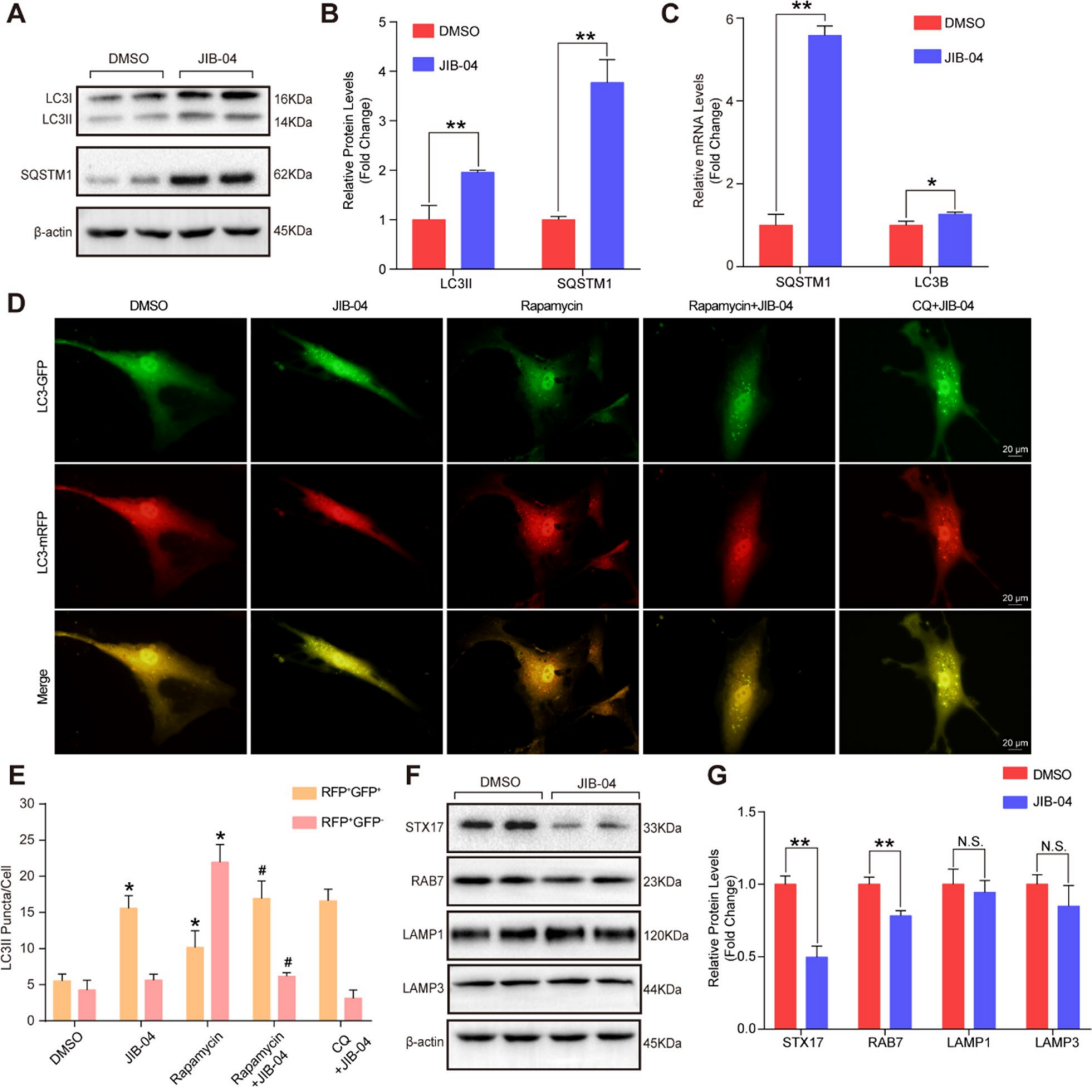
JIB-04 inhibited autophagic flux by regulating STX17 and RAB7 to disturb the fusion of autophagosomes and lysosomes. **A-B**. Western blotting was used to evaluate the protein levels of SQSTM1 and LC3 in HASMCs treated with JIB-04 (0.5 μM) for 48 h (*n* = 4 per group). **C**. Quantification of the mRNA levels of SQSTM1 and LC3B after treatment with JIB-04 (0.5 μM) for 48 h (*n* = 3 per group). **D-E**. HASMCs were transfected with lentivirus harboring tandem fluorescent mRFP-GFP-LC3 to monitor autophagy. Representative images of immunofluorescent VSMCs are shown. Autophagosomes are yellow dots, and autophagolysosomes are red dots. Scale bar: 20 μm. Quantitative analysis of red and yellow dots numbers. *P < 0.05 versus the DMSO group; #*P* < 0.05 versus the Rapamycin group. **F-G**. Western blot analysis showed the protein expression levels of STX17, RAB7, LAMP1, and LAMP3 (*n* = 4 per group). β-Actin served as a loading control. *P < 0.05, **P < 0.01, N.S. no significant

Intriguingly, in addition to cell cycle- and mitosis-related pathways, autophagy was also enriched according to Gene Set Enrichment Analysis (GSEA) and Kyoto Encyclopedia of Genes and Genomes (KEGG) (Fig. 6A and B). The differentially expressed genes enriched in the autophagy pathway are presented as a heatmap in Fig. 6C. In addition, the PI3K-AKT signaling pathway and mTOR signaling pathway were notably enriched in HASMCs after treatment with JIB-04 (Fig. 6B), and further validation showed that the phosphorylation of AMPKα was markedly increased, while the phosphorylation of mTOR was decreased in the HASMCs treated with JIB-04 (Fig. 6D and E). Given that these pathways were involved in the response to amino acid starvation and glucose starvation (Fig. 6F) and that both amino acid starvation and glucose starvation can induce robust autophagy in cells [8], we further detected the expression levels of autophagy-related molecules. Our results demonstrated that compared with DMSO treatment, JIB-04 treatment increased the protein levels of LC3II and SQSTM1 (Fig. 7A–C). SQSTM1 is a critical protein in autophagy that is considered to mediate selective autophagy [20]. There may be two reasons for the increase in the SQSTM1 protein level: one is increased protein synthesis, and the other is the accumulation caused by the blocked autophagic degradation pathway. Thus, to clarify the reason for the elevated SQSTM1, we tracked autophagic flux with the autophagy double-labeled lentivirus (mRFP-GFP-LC3) in the HASMCs with the indicated treatments (Fig. 7D). The results showed that more orange puncta were observed in the JIB-04-treated HASMCs compared with that of the DMSO group, and CQ did not further increase the number of orange puncta (Fig. 7D and E). Rapamycin treatment significantly promoted autophagosome formation and enhanced autophagic flux, as evidenced by more red puncta, while treatment with rapamycin combined with JIB-04 resulted in a significant accumulation of orange puncta but not red puncta (Fig. 7D and E). Since GFP should be degraded and quenched after autophagosome-lysosome fusion, these results suggested that JIB-04 strikingly inhibited autophagosome-lysosome fusion, resulting in increased orange puncta. Furthermore, we further detected key regulators, including STX17, RAB7, LAMP1, and LAMP3, involved in the fusion of autophagosomes and lysosomes, and found that STX17 and RAB7 were markedly downregulated by JIB-04, while comparable LAMP1 and LAMP3 protein levels were detected between the groups (Fig. 7F and G), which indicated that JIB-04 might inhibit the fusion of autophagosomes and lysosomes and impair autophagic flux by reducing STX17 and RAB7 expression.

## Discussion

**Fig. 8:**
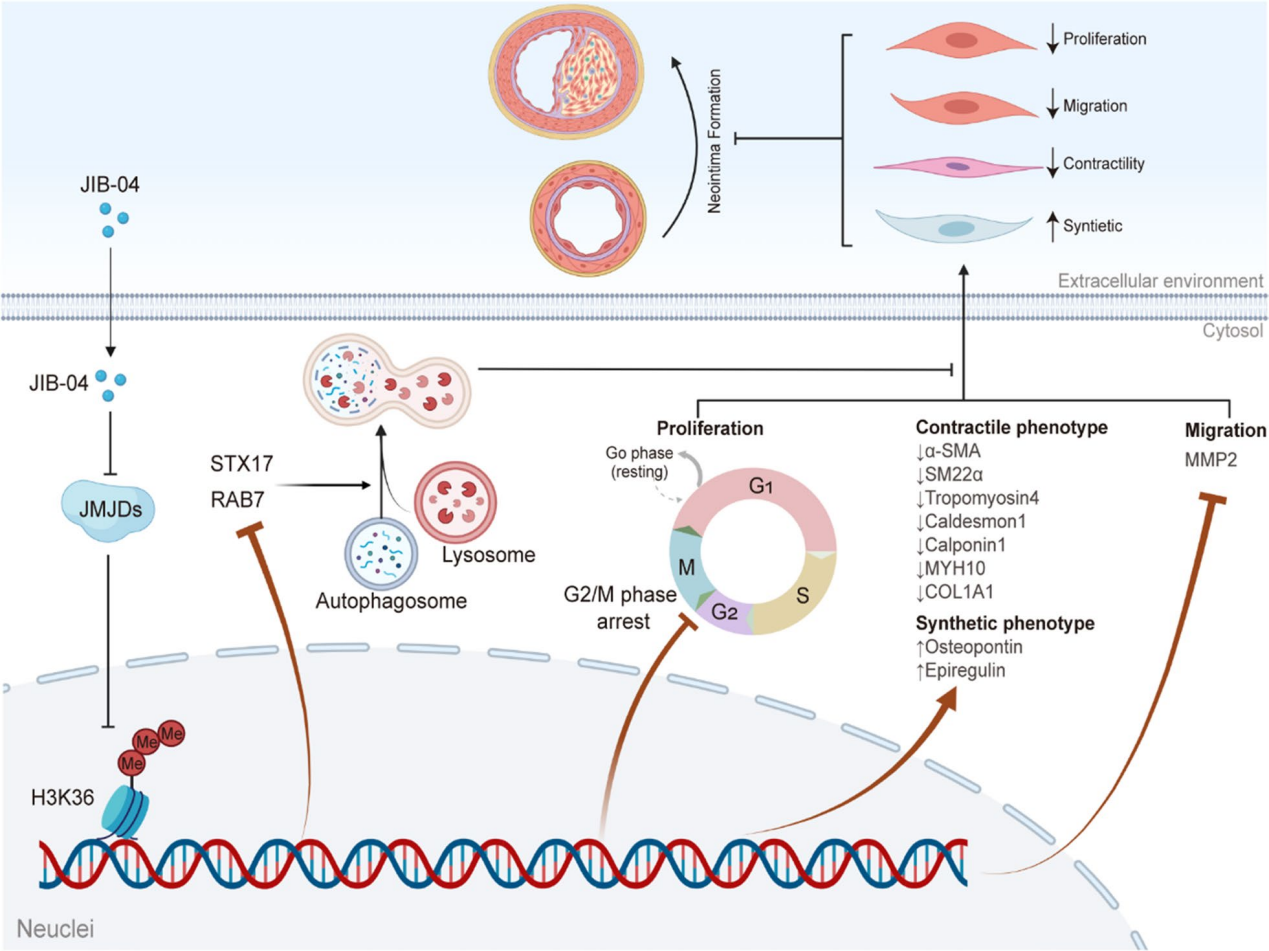
JIB-04 inhibited the proliferation, migration, and contractile phenotype of HASMCs by blocking autophagic flux and probably regulated the neointima. JIB-04, an inhibitor of Jumonji C domain-containing demethylases (JMJDs), induced cell cycle arrest in G2/M phase and inhibited the proliferation, migration, and contractile phenotype of HASMCs by regulating the methylation of H3K36. In addition, JIB-04 inhibited the fusion of autophagosomes and lysosomes by downregulating STX17 and RAB7, which further restrained the proliferation, migration, and contractile phenotype of HASMCs, ultimately protecting against neointima formation. Created by Biorender.com

In recent years, epigenetic modification has emerged as a promising target for cardiovascular disease treatment [6, 21]. As a critical part of epigenetic modification, protein methylation participates in the regulation of several kinds of vascular diseases, including neointima formation [6]. In this study, we first revealed that JIB-04, a pan-inhibitor of Jumonji C domain demethylases, could arrest HASMCs at the G2/M checkpoint and impair autophagic flux to inhibit HASMC proliferation, migration, and contractile phenotypes by regulating the methylation of H3K36 (Fig. 8), which indicated that JIB-04 may be a potential drug for preventing neointima formation.

In the primary cultured HASMCs treated with different concentrations of FBS to induce proliferation, we found that the levels of H3K36me3, H3K9me3, and H3K4me3 were downregulated. Furthermore, in the mouse model of neointima formation, lower levels of H3K36me3 and H3K4me3, but a higher level of H3K9me3 in the injured carotid arteries than in normal controls. These results suggested that there is a paradox of H3K9 methylation on proliferation in vivo and in vitro, or the function of H3K9 methylation is stimulation-dependent. In the HASMCs treated with JIB-04, the methylation levels of H3K36 and H3K9, but not H3K4 were upregulated, which indicated that JIB-04 only increases H3K36 and H3K9 methylation levels in HASMCs. Based on these results, we were prudent to conclude that JIB-04 regulates phenotypic switching of VSMCs by upregulating the methylation of H3K36.

Neointima formation is mainly caused by excessive proliferation of VSMCs, and cell cycle checkpoints control cell division and proliferation [22]. In general, the cell cycle is primarily regulated by cyclin, cyclin-dependent kinases (CDKs), and CDK inhibitors (CKIs) [23, 24]. In the present study, we found that JIB-04 arrested HASMCs at the G2/M checkpoint to inhibit proliferation. It has been reported that CDK1 (also known as CDC2)-cyclinB1 complexes regulate G2/M phase progression and trigger mitosis [25]. WEE1 catalyzes the inhibitory tyrosine phosphorylation of CDK1/cyclin B kinase to inhibit cell entry into mitosis [26]. In contrast, CDC25C was reported to rapidly remove CDK1 phosphorylation on Tyr15/Thr14 to increase CDK1 activity and then trigger entry into mitosis [26]. In accordance with the reported results, we found that JIB-04 suppressed the phosphorylation of CDC2 and Rb and downregulation of CDC25C but upregulated WEE1 expression, resulting in G2/M phase arrest.

Given that inhibition of VSMC proliferation is often accompanied by altered migratory capacity, we demonstrated that JIB-04 also inhibited the migration of HASMCs by downregulating the expression of MMP2 but not MMP9. MMP2 is a zinc-dependent enzyme cleaving components of the extracellular matrix to facilitate cell migration [27]. In addition, the switching of VSMCs from a contractile to a synthetic phenotype was associated with their proliferation [17]. The MAPK signaling pathway and PI3K-AKT signaling pathway play an important role in modulating VSMC phenotype switching [28]. Zhang et al. revealed that serum amyloid A (SAA) induced downregulation of contractile gene expression and upregulation of synthetic gene expression, while a P38 inhibitor reversed the phenotype switching from a contractile phenotype to a synthetic phenotype caused by SAA [29]. Moreover, AKT activation was identified to promote PDGF-induced phenotype switching in SMCs [30]. Our results showed that compared with DMSO, JIB-04 facilitated the phenotypic switching of HASMCs from contractile to synthetic by promoting the phosphorylation of AKT, FOXO3A, and P38 in HASMCs.

Autophagy is a highly conserved catabolic process for the preservation of cellular and organismal homeostasis [31]. Autophagy is a multistep cellular biological process that includes the initiation and formation of autophagosomes, autophagolysosomes, and degradation [32]. Although autophagy was originally considered a kind of cellular self-protection, abnormal formation of autophagosomes or the degradation of contents may have adverse effects on cells [7, 8]. In this study, RNA-sequencing analysis indicated that JIB-04 not only regulated HASMC proliferation but also affected autophagy. Autophagy is closely related to the function of VSMCs; for example, the pharmacological inhibition of autophagic formation by 3-MA prevented PDGF-induced VSMC proliferation [33]. Notably, disruption in different phases of autophagy may have different effects on cells. We found that JIB-04 upregulated the expression of the autophagy-related proteins LC3II and SQSTM1. As both autophagic activation and impaired autophagic flux may lead to elevated protein levels of LC3II and SQSTM1, to investigate the reasons for their upregulation, we monitored the autophagic process by mRFP-GFP-LC3 labeling in combination with CQ. Our results demonstrated that JIB-04 impaired autophagic flux by inhibiting the expression of STX17 and RAB7. STX17, a soluble N-ethylmaleimide-sensitive factor-attachment protein receptor (SNARE) of the autophagosome, interacts with cytosolic SNAP29 and lysosomal VAMP8 to facilitate autophagosome-lysosome fusion [34]. RAB7 is also involved in autophagosome maturation by promoting microtubule plus-end-directed transport and fusion of autophagosomes with lysosomes [35]. Increasing evidence has indicated that the accumulation of nonfused autophagosomes exacerbates cytotoxicity and inhibits cell proliferation [36, 37]. As JIB-04 suppressed the expression of STX17 and JIB-04, we cautiously concluded that JIB-04 impaired autophagic flux by blocking the fusion of autophagosomes with lysosomes to suppress proliferation.

## Conclusion

In conclusion, we demonstrated that JIB-04, a pan-inhibitor of JMJD demethylases, inhibited the proliferation and migration of HASMCs by arresting cells at G2/M checkpoints and inhibiting MMP2 expression, respectively. However, JIB-04 also downregulated the expression of STX17 and RAB7 to disrupt the fusion of autophagosomes with lysosomes. Our results suggested that JIB-04 can affect the function of VSMCs by regulating proliferation and autophagy and is expected to be a candidate drug for the prevention or treatment of neointima formation.

## Methods and materials

### Animal experiments

All animal experiments were performed in accordance with the protocols approved by the Animal Care and Use Committees of Tongji Hospital, Tongji Medical College, Huazhong University of Science and Technology. Male C57BL/6 mice were housed in specific-pathogen-free facilities with a 12-h light/dark cycle and controlled temperature (20–22 °C). The carotid artery injury model was established as reported previously [17]. Briefly, adult male mice were assigned randomly to the sham and injury groups. After mice were anesthetized by intraperitoneal (IP) injection of 3% pentobarbital sodium, a 4–5 mm ventral midline incision was made in the neck, and the left carotid artery (CA) was exposed. Subsequently, the external CA was tied off with an 8–0 suture near the bifurcation point. The left common carotid artery and internal carotid artery were interrupted by hemostatic clamping. A metal guide wire (0.38 mm in diameter, No. C-SF-15–15; Cook, Bloomington, USA) was advanced 1 cm through transverse arteriotomy of the external CA. Endothelial denudation was achieved by advancing and withdrawing the guidewire five times with a rotating motion along the common CA. Then, the guidewire and clamps were removed after injury. Muscle layers and skin were closed with 6–0 silk sutures. The same procedure without incision and artery injury was performed in sham littermate control mice. Both injured and uninjured carotid artery tissues were obtained on the 28th day after endothelial denudation.

### Histological analysis and immunohistochemistry assay

Histological analysis and immunohistochemistry were performed as reported previously [38]. In short, mouse carotid artery tissues were placed into 4% formalin for fixation after being washed with a cold saline solution. Tissues were dehydrated and embedded in paraffin after fixation for 3 days. Embedded carotid artery tissues were sectioned into 5- to 10-μm-thick cross slices. H&E staining was performed to observe whether the thickness of the intima layer was increased in the injured carotid artery. Immunohistochemistry assays were performed as described previously [39]. Briefly, tissue slices were first deparaffined with xylene and hydrated with gradient ethanol. Then, cross slices were submerged in citrate solution (pH 6.0) and boiled at 100 degrees centigrade for antigen retrieval, followed by incubation in 3% H_2_O_2_. Tissue sections were blocked with 5% BSA after washing with phosphate-buffered saline (PBS). Primary antibodies against H3K36me3 were used to incubate sections at 4 ℃ overnight, followed by incubation with secondary antibodies for 20 min. Afterward, the sections were stained with diaminobenzidine (DAB) and counterstained with hematoxylin. Sections were then observed after dehydration with gradient ethanol and vitrification with xylene.

### Cell cultivation and treatment

HASMCs were extracted from aortic tissue trimmed from heart donors during transplantation surgery, and HASMCs were cultivated as reported previously [40]. In brief, ascending aortic tissues were taken from patients who underwent heart transplantation. Then, tissues were placed into culture dishes filled with Dulbecco's modified Eagle's medium (DMEM)/F12 (SH30023.01; HyClone) at 4 °C. Intimal and residual adventitial tissues were stripped under a stereomicroscope. Subsequently, the media of the vessels was cut into small pieces (1–2 mm) and transferred to cell culture flasks with 5 mL of DMEM/F12 with 10% fetal bovine serum (SH30084.03; HyClone) and 1% penicillin–streptomycin (15,140–122; Thermo Fisher Scientific). A few days later, long spindle smooth muscle cells were observed and then passaged when the degree of cell fusion reached approximately 80%. HASMCs were separated from human ascending aortic tissue and cultured with DMEM/F12 with 10% fetal bovine serum and 1% penicillin–streptomycin. All cells were cultured at 37 °C in a humidified incubator with 5% CO_2_.

### CCK-8 assay

A CCK-8 (Cell Counting Kit-8, BS350A; Biosharp) assay was performed to measure the toxicity of JIB-04 on HASMCs and the effect of JIB-04 on HASMC proliferation. For determination of the toxicity of JIB-04, a total of 8 × 10^3^ cells/100 μL per well were plated in 96-well plates and treated with a JIB-04 concentration gradient (0, 0.1, 0.25, 0.5, 1 μmol/L) for 24 h. CCK-8 reagent (10 μL per well) was added to DMEM per well and incubated at 37 °C for 1.5 h, and the absorbance at 450 nm was measured using a BioTek Synergy HT microplate reader. For measurement of the effect of JIB-04 on proliferation, a total of 4 × 10^3^ cells/100 μL per well were plated in 96-well plates and treated with DMSO or 0.5 μM JIB-04 for 0 h, 24 h, 48 h, or 72 h. Then, CCK-8 reagent was added, and 450 nm absorbance was detected as described above.

### LDH assay

A lactate dehydrogenase (LDH) assay was performed to evaluate the toxicity of JIB-04 on HASMCs by a Cytotoxicity LDH Assay Kit-WST (CK12, Dojindo) as reported previously [41]. A total of 8 × 10^3^ cells/100 μL per well were plated in 96-well plates, which were treated with a JIB-04 concentration gradient (0, 0.1, 0.25, 0.5, 1 μmol/L) for 24 h. Then, 10 μL of lysis buffer was added to the high contrast well and incubated at 37 °C for 30 min. Then, 100 μL working solution was added to each well and cultivated at room temperature in the dark for 15 min. Next, 50 μL of stop solution was added to each well, and the absorbance at 490 nm was measured using a BioTek Synergy HT microplate reader.

### EdU incorporation assay

A Cell-Light™ Edu Apollo 567 In Vitro kit (C10310-1, RiboBio) was used to perform the EdU incorporation assay. HASMCs treated with DMSO or 0.5 μM JIB-04 were plated in 24-well plates at 2 × 10^4^ cells per well and incubated with EdU medium (50 µmol/L) made up of EdU solution and complete medium at a ratio of 1:1000 for 4 h. Cells were fixed with 4% paraformaldehyde for 30 min after washing with by PBS. Then, the cells were incubated with glycine (2 mg/mL) to neutralize paraformaldehyde and were incubated with a penetrant made up of PBS and 0.5% Triton X-100 for 10 min. Cells were colored with 1 × Apollo staining solution and then DNA was stained with DAPI after the incubation with 0.5% Triton X-100 PBS solution again. The cells were washed with PBS and observed by fluorescence microscopy. Images were captured by an Olympus light microscope BX53 system.

### Immunofluorescence staining

After cultivation and treatment with DMSO or 0.5 μM JIB-04 on cell climbing slices for 24 h, HASMCs were fixed with 4% paraformaldehyde for 30 min, followed by penetrating the cell membrane with 0.5% Triton X-100 for 30 min. Then, the samples were blocked by using 5% BSA for 60 min at room temperature. Subsequently, the primary antibodies against Ki67 (ab16667, 1:200 dilution) and α-SMA (ab7817, 1:100 dilution) were incubated overnight at 4 °C. The next day, the Alexa Fluor 568 donkey anti-rabbit IgG (H + L) (Thermo Fisher Scientific, A10042, 1:200 dilution) secondary antibody and Alexa Fluor 488 donkey anti-mouse IgG (H + L) (Thermo Fisher Scientific, A10042, 1:100 dilution) secondary antibody were incubated in Ki67 and α-SMA cell climbing slices, respectively, for 60 min. DAPI was used to stain nuclei after washing with PBS three times. An Olympus light microscope BX53 system was applied for image capture.

### Flow cytometry

HASMCs were collected by trypsin digestion, followed by washing twice with phosphate-buffered saline (PBS). Cells were collected by centrifugation for 10 min. After the cells were resuspended with PBS, 70% ethanol precooled at − 20 °C was added to fix the cells at 4 °C overnight. The next day, the cells were washed with PBS twice after discarding ethanol. Then, 150 μL of Ribonuclease A (R5125; Sigma-Aldrich) and 150 μL of PI (P4864; Sigma- Aldrich) were applied to stain cells for 4 h in darkness. The stained cells were sorted by using a BD FACSAria™ III sorter, and Flow Jo software (version 10.7.2) was selected for cell cycle analysis.

### Transwell migration assay

After treatment with DMSO or JIB-04 (0.5 μM) for 24 h, HASMCs were collected by trypsin digestion and resuspended in DMEM/F12 with 0.5% fetal bovine serum. HASMCs (2.5 × 10^4^ cells/100 μL per well) were seeded onto transwell inserts with a polyethylene terephthalate membrane pore size of 8 μm (3422, Corning) in 24-well plates. Transwell inserts were transferred and submerged in 800 μL of DMEM/F12 with 10% fetal bovine serum after adherence for 2 h. After migration for 12 h, media within the transwell inserts were carefully removed. Cells were fixed with 4% paraformaldehyde and stained with crystal violet (Sigma-Aldrich). Cells that did not migrate across the transwell membrane were then removed by gentle wiping with a cotton swab. Migrated cells were viewed with an inverted microscope (Nikon).

### RNA sequencing (RNA-seq) and analysis

For RNA sequencing, HASMCs were treated with DMSO (DMSO group) or 0.5 μM JIB-04 (JIB-04 group). After treatment for 48 h, the RNA of HASMCs was extracted and processed by using an RNA-seq pipeline by Novogene Co., Ltd. (Beijing, China). The RNA integrity was evaluated by the RNA Nano 6000 Assay Kit of the Bioanalyzer 2100 system (Agilent Technologies, CA, USA). After quantitative processing, the count files were used as input to the R (version 4.1.2) package DEseq2, and the differentially expressed genes were analyzed based on the negative binomial distribution model. To improve accuracy, we adopted Benjamini and Hochberg’s approach to adjust P value. We screened the differentially expressed genes by an absolute value of logfold change greater than 0.7 and an adjusted P value less than 0.05. Based on the differentially expressed genes, KEGG, GO, and GSEA were performed by the clusterProfiler (v3.16.1) R package [42].

### Autophagic flux assay

For monitoring autophagic flux, mRFP-GFP-LC3 lentivirus packed with the mRFP-GFP-LC3 plasmid was used to mark and track LC3. HASMCs were transfected with LC3-mRFP-GFP lentivirus for 24 h. For synchronization, HASMCs were cultivated with DMEM/F12 without fetal bovine serum for 12 h. Then, HASMCs were incubated with JIB-04 (0.5 μM) for 48 h, rapamycin (150 nM) for 12 h and CQ (20 μM) for 12 h. Images were observed under an Olympus light microscope BX53 system. GFP was quenched when the pH decreased, while mRFP remained stable. Thus, after merging the green and red fluorescence images, the yellow puncta were considered autophagosomes (GFP^+^/RFP^+^), while the red puncta were considered autophagolysosomes (GFP^−^/RFP^+^). The autophagic flux was assessed by counting the number of yellow and red puncta.

### Antibodies

The antibodies used in this study included α-SMA (ab7817), SM22α (ab14106), H3K9me1 (ab9045), H3K9me2 (ab1220), H3K4me1 (ab8895), H3K36me2 (ab9049), H3K36me3 (ab9050), which were bought from Abcam. β-actin (AC026) and RAB7 (A12308) were got from ABclonal. H3K9me3 (GTX121677), MMP2 (GTX634832), MYH10 (GTX634160), MMP9 (GTX100458), PCNA (GTX100539) were purchased from GeneTex. P-H3 (sc-8656-R) was obtained from Santa Cruz. LAMP3 (AP1827A) was bought from Abgent. STX17 (HPA001204) was got from ATLAS. H3K4me2 (#9725), H3K4me3 (#9727), H3K36me1 (#14,111), p-AKT (#4060), AKT (#4685), p-FOXO3 (#9466), FOXO3A (#12,829), p-P38 (#4511), P38(#8690), p-CDC2 (#4539), p-Rb (#8516), Rb (#9313), p-CHK1(#2348), P-CHK2 (#2197), LC3A/B (#12,741), SQSTM1 (#88,588), p-AMPKα (#5831), AMPKα (#2535), p-mTOR (#5536), mTOR (#2983), LAMP1 (#9091), and COL1A1 (#91,144) were purchased from Cell Signaling Technology.

### Western blot

Total proteins derived from HASMCs were extracted by a complete cell lysis solution made up of RIPA and PMSF at a ratio of 100 to 1. Ultrasound and 95 °C high temperature were used to denaturant proteins. After denaturation, equal amounts of protein were subjected to sodium dodecyl sulfate-polyacrylamide gel electrophoresis and then transferred to a polyvinylidene fluoride membrane (Millipore, IPVH00010), which was blocked with 5% skim milk in Tris Tween-buffered saline. After that, the membrane was incubated with the indicated primary antibody overnight at 4 °C and the peroxidase-conjugated secondary antibody (Jackson ImmunoResearch Laboratories, 111–035-003, at 1:10,000 dilution) for 1 h at room temperature. Protein bands were detected by using the ChemiDoc ™ XRS + system (Bio-Rad).

### Real-time PCR

Total mRNA was extracted from humans by using TRI Reagent® Solution (AM9738; Thermo Fisher Scientific). The precipitated mRNA was dissolved in nuclease-free water, and the RNA concentration was determined by a Nanodrop2000 (Thermo Fisher Scientific). Subsequently, mRNA was transcribed into cDNA by using a Transcriptor First Strand cDNA Synthesis Kit (4,896,866,001, Roche). Then, 2 μL of DNA solution, primers, DEPC water, and Hieff® qPCR SYBR® Green Master Mix (Yeasen, 11201ES08) were added to each well for the real-time PCR assay. Furthermore, the relative mRNA levels were detected by a CFX Connect™ Real-Time PCR Detection System (Bio-Rad) using iQ™SYBR ® Green Supermix (1,708,884; Bio-Rad). The primers used in this study are listed in Additional file 3: Table S1.

### Statistical analysis

All the data are represented as the mean ± standard deviation (SD) in the present study. Statistical analysis was carried out by using Student’s two-tailed *t* test to compare the means of two groups, while multiple group comparisons were made by using one-way ANOVA with least significant difference (equal variances assumed) or Tamhane T2 (equal variances not assumed) tests in SPSS software (version 23.0). *P* < 0.05 was considered statistically significant.

## Supplementary Information

**Additional file 1: Figure S1.** The expression of H3K9 and H3K4 correlated with HASMC proliferation and neointima formation.

**Additional file 2: Figure S2.**  JIB-04 treatment regulated the methylation level of H3K9 rather than H3K4 in HASMCs.

**Additional file 3: Table S1.**  Primers for Real-Time PCR detection.

## Data Availability

All data generated or analyzed during this study are included in this published article and its supplementary information files. RNA sequencing data for this study will be provided from the corresponding author on reasonable request.

## Abbreviations

CVDs: Cardiovascular diseases
CHD: Coronary atherosclerotic heart disease
PCIs: Percutaneous coronary interventions
CABG: Coronary artery bypass grafting
CVDs: Cardiovascular diseases
VSMC: Vascular smooth muscle cell
HASMCs: Human aortic smooth muscle cells
JMJD: Jumonji C domain-containing protein
KDMs: Histone lysine demethylases
CA: Carotid artery
FBS: Fatal bovine serum
PBS: Phosphate-buffered saline
DMEM: Dulbecco's modified Eagle's medium
CCK-8: Cell Counting Kit-8
LDH: Lactate dehydrogenase
PI: Propidium iodide
EDTA: Ethylenediaminetetraacetic acid
SDS: Sodium dodecyl sulfate
CQ: Chloroquine
GFP: Green fluorescent protein
RFP: Red fluorescent protein
PMSF: Phenylmethylsulfonyl fluoride
DEPC: Diethyl pyrocarbonate
MAPK: Mitogen-activated protein kinase
PCA: Principal component analysis
BP: Biological process
GO: Gene Ontology
CC: Cellular component
KEGG: Kyoto Encyclopedia of Genes and Genomes
GSEA: Gene Set Enrichment Analysis
CDKs: Cyclin-dependent kinases
CKIs: CDK inhibitors
OPN: Osteopontin
MGP: Matrix Gla protein
