# Supplementary material for: JIB-04, a histone demethylase Jumonji C domain inhibitor, regulates phenotypic switching of vascular smooth muscle cells
Source: Clin Epigenetics. 2022 Aug 13;14:101. doi: 10.1186/s13148-022-01321-8 (PMC9375951; doi:10.1186/s13148-022-01321-8)
Supplement: Supplementary file 1 — Additional file 1: Figure S1. The expression of H3K9 and H3K4 correlated with HASMC proliferation and neointima formation. [file 13148_2022_1321_MOESM1_ESM.docx]

**Supplemental Figure**

**Figure S1**


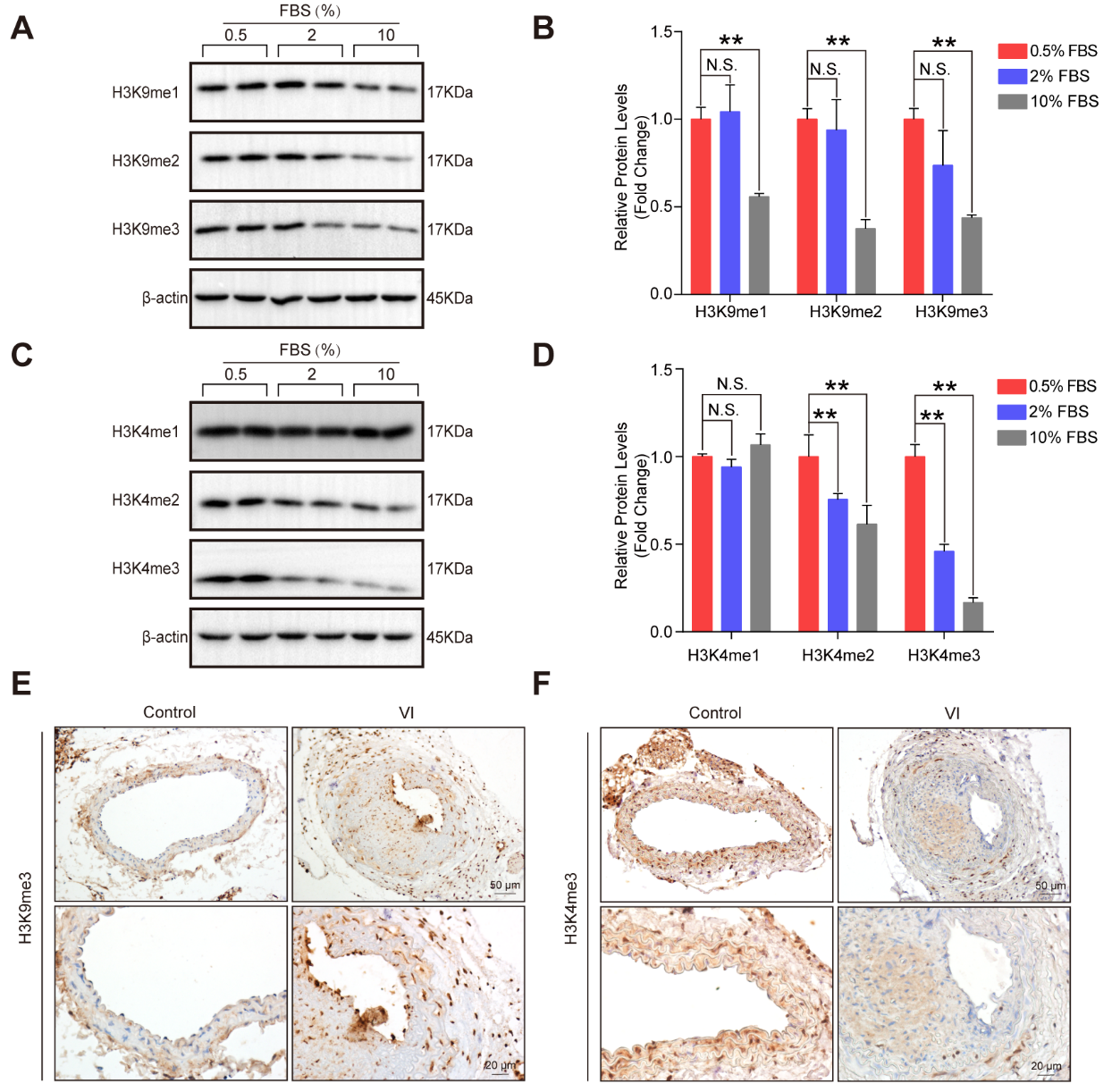


**Figure S1. The expression of H3K9 and H3K4 correlated with HASMC proliferation and neointima formation. (A-B).** Western blot analysis showed the protein expression of H3K9me1, H3K9me2 and H3K9me3 in HASMCs cultivated with 0.5%, 2% and 10% fetal bovine serum (FBS) (n=4 per group). β-Actin served as a loading control. **P*<0.05, ***P*<0.01. N.S. no significant. (**C-D).** Western blot analysis showed the protein expression of H3K4me1, H3K4me2 and H3Kme3 in HASMCs cultivated with 0.5%, 2% and 10% fetal bovine serum (FBS) (n=4 per group). β-Actin served as a loading control. **P*<0.05, ***P*<0.01. N.S. no significant. **(E).** Representative images of immunohistochemical staining of H3K9me3 in the carotid arteries. H3K9me3-labeled nuclei are shown in brown. Scale bar: 20 and 50 μm. (**F).** Representative images of immunohistochemical staining of H3K4me3 in the carotid arteries. H3K4me3-labeled nuclei are shown in brown. Scale bar: 20 and 50 μm.
