# Supplementary material for: JIB-04, a histone demethylase Jumonji C domain inhibitor, regulates phenotypic switching of vascular smooth muscle cells
Source: Clin Epigenetics. 2022 Aug 13;14:101. doi: 10.1186/s13148-022-01321-8 (PMC9375951; doi:10.1186/s13148-022-01321-8)
Supplement: Supplementary file 2 — Additional file 2: Figure S2. JIB-04 treatment regulated the methylation level of H3K9 rather than H3K4 in HASMCs. [file 13148_2022_1321_MOESM2_ESM.docx]

**Supplemental Figure**

**Figure S2**


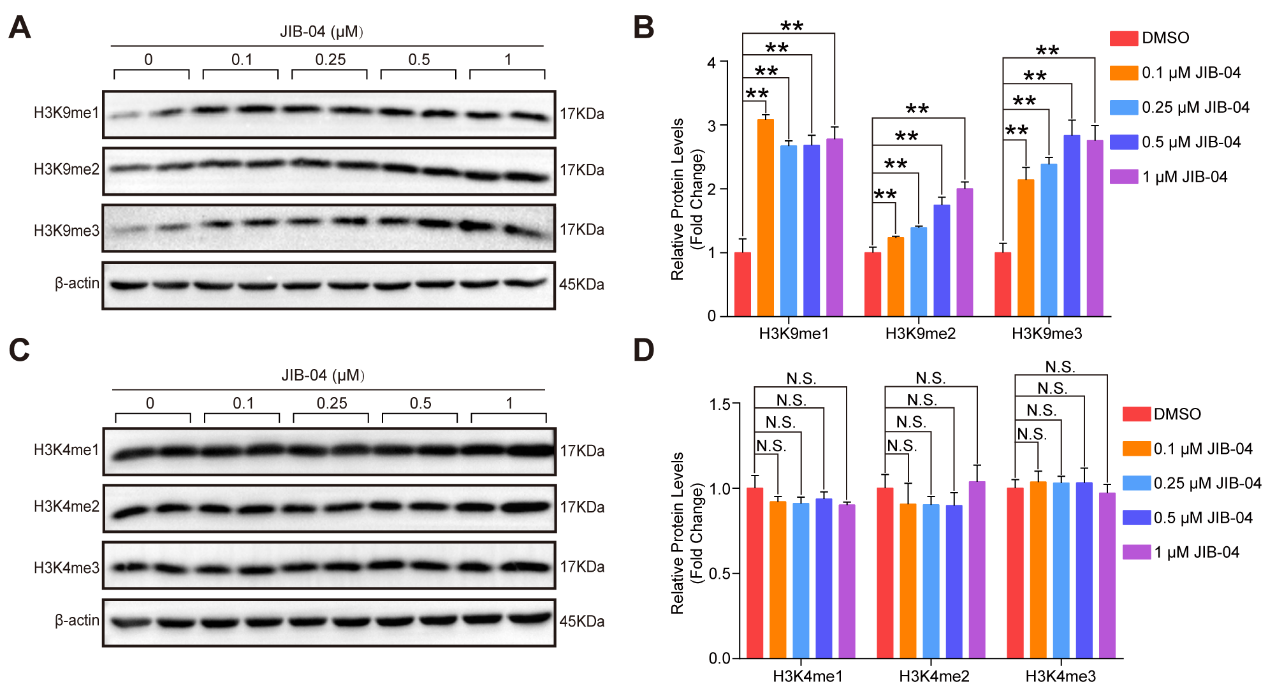


**Figure S2.** **JIB-04 treatment regulated the methylation level of H3K9 rather than H3K4 in HASMCs. (A-B)**. H3K9me1, H3K9me2 and H3K9me3 protein levels in HAMSCs treated with different concentrations of JIB-04 (0, 0.1, 0.25, 0.5, 1 μM) were measured by western blotting (n=4 per group). (**C-D**). H3K9me1, H3K9me2 and H3K9me3 protein levels in HAMSCs treated with different concentrations of JIB-04 (0, 0.1, 0.25, 0.5, 1 μM) were measured by western blotting (n=4 per group). β-Actin served as a loading control. *P<0.05, **P<0.01, N.S. no significant.
