# Supplementary material for: JIB-04, a histone demethylase Jumonji C domain inhibitor, regulates phenotypic switching of vascular smooth muscle cells
Source: Clin Epigenetics. 2022 Aug 13;14:101. doi: 10.1186/s13148-022-01321-8 (PMC9375951; doi:10.1186/s13148-022-01321-8)
Supplement: Supplementary file 3 — Additional file 3: Table S1. Primers for Real-Time PCR detection. [file 13148_2022_1321_MOESM3_ESM.docx]

**Supplemental Table**

**Table S1. Primers for Real-Time PCR detection.**

| Gene | Sequence | |
| --- | --- | --- |
| CCNB1 | Forward | 5’-ATGCAGCACCTGGCTAAGAA-3’ |
|  | Reverse | 5’-TACACCTTTGCCACAGCCTT-3’ |
| CCNB2 | Forward | 5’-GCTCCAAAGGGTCCTTCTCC-3’ |
|  | Reverse | 5’-ACCTCCAGCTGCCTGAGATA-3’ |
| CDK1 | Forward | 5’-CTGGGGTCAGCTCGTTACTC-3’ |
|  | Reverse | 5’-TCCACTTCTGGCCACACTTC-3’ |
| CCNA1 | Forward | 5’-TACCTCAAAGCACCACAGCA-3’ |
|  | Reverse | 5’-TGGGTTGAGGAGAGAAACACC-3’ |
| CDC25C | Forward | 5’-TGGCGGAAGGTTTGAATGGT-3’ |
|  | Reverse | 5’-CCTGAGCAGAAGGCCAAAGT-3’ |
| PLK1 | Forward | 5’-TTCCACGGCTTTTTCGAGGA-3’ |
|  | Reverse | 5’-CCCAGCTTGAGGTCTCGATG-3’ |
| WEE1 | Forward | 5’-GGGCGATAGTCGTTTTCTTGC-3’ |
|  | Reverse | 5’-CACATACCACTGTGAGGGCA-3’ |
| α-SMA | Forward | 5’-CAGCTACGTGGGTGACGAAG-3’ |
|  | Reverse | 5’-ATGCTCTTCAGGGGCAACAC-3’ |
| MYH10 | Forward | 5’-TGGTCTTCATGAGCCACCAGT-3’ |
|  | Reverse | 5’-AACGGTACGAAACATGCCCT-3’ |
| Tropomyosin4 | Forward | 5’-GTGCGGAGGTGTCTGAACTA-3’ |
|  | Reverse | 5’-GCCTGGGCAAGTTTCTCTTC-3’ |
| COL1A1 | Forward | 5’-GAGGGCCAAGACGAAGACATC-3’ |
|  | Reverse | 5’-CAGATCACGTCATCGCACAAC-3’ |
| Calponin1 | Forward | 5’-TTAAGAACAAGCTGGCCCAGAAG-3’ |
|  | Reverse | 5’-TGAAGTTGCCGATGTTCTCCA-3’ |
| Caldesmon1 | Forward | 5’-AAACAGTCACCAAGTCCTACCA-3’ |
|  | Reverse | 5’-ACTGTTTCCTCCTGGCTTTCA-3’ |
| Fibronectin | Forward | 5’-AGGAGAACAGTGGCAGAAGG-3’ |
|  | Reverse | 5’-GGGAATCTTCTCTGTCAGCC-3’ |
| MGP | Forward | 5’-TTTGTGTTATGAATCACATGAAAGC-3’ |
|  | Reverse | 5’-AGCGTTCTCGGATCCTCTCT-3’ |
| OPN | Forward | 5’-CAGCTTTACAACAAATACCCAGATG-3’ |
|  | Reverse | 5’-GACTTACTTGGAAGGGTCTGTG-3’ |
| Epiregulin | Forward | 5’-CTGCCTGGGTTTCCATCTTCT-3’ |
|  | Reverse | 5’-ATTGACACTTGAGCCACACG-3’ |
| SQSTM1 | Forward | 5’-ATGGCCATGTCCTACGTGAAG-3’ |
|  | Reverse | 5’-GCCATCGCAGATCACATTGG-3’ |
| LC3B | Forward | 5’-CCGACTTATTCGAGAGCAGCA-3’ |
|  | Reverse | 5’-AGCTGCTTCTCACCCTTGTATC-3’ |
| ATG5 | Forward | 5’-GCTTCGAGATGTGTGGTTTGG-3’ |
|  | Reverse | 5’-CCATTTCAGTGGTGTGCCTTC-3’ |
| ATG7 | Forward | 5’-GCATCCAGAAGGGGGCTATG-3’ |
|  | Reverse | 5’-GATCAAGAACCTGGTGAGGCA-3’ |
| BECN1 | Forward | 5’-CAGGAGAGACCCAGGAGGAA-3’ |
|  | Reverse | 5’-GCTGTTGGCACTTTCTGTGG-3’ |
| 18S | Forward | 5’-CTCAACACGGGAAACCTCAC-3’ |
|  | Reverse | 5’-CGCTCCACCAACTAAGAACG-3’ |
